# Identification of motor progression in Parkinson’s disease using wearable sensors and machine learning

**DOI:** 10.1038/s41531-023-00581-2

**Published:** 2023-10-07

**Authors:** Charalampos Sotirakis, Zi Su, Maksymilian A. Brzezicki, Niall Conway, Lionel Tarassenko, James J. FitzGerald, Chrystalina A. Antoniades

**Affiliations:** 1 NeuroMetrology Lab, Nuffield Department of Clinical Neurosciences, University of Oxford, Oxford, UK; 2 Institute of Biomedical Engineering, Department of Engineering Science, University of Oxford, Oxford, UK; 3 Nuffield Department of Surgical Sciences, University of Oxford, Oxford, UK

**Keywords:** Parkinson's disease, Predictive markers

## Abstract

Wearable devices offer the potential to track motor symptoms in neurological disorders. Kinematic data used together with machine learning algorithms can accurately identify people living with movement disorders and the severity of their motor symptoms. In this study we aimed to establish whether a combination of wearable sensor data and machine learning algorithms with automatic feature selection can estimate the clinical rating scale and whether it is possible to monitor the motor symptom progression longitudinally, for people with Parkinson’s Disease. Seventy-four patients visited the lab seven times at 3-month intervals. Their walking (2-minutes) and postural sway (30-seconds,eyes-closed) were recorded using six Inertial Measurement Unit sensors. Simple linear regression and Random Forest algorithms were utilised together with different routines of automatic feature selection or factorisation, resulting in seven different machine learning algorithms to estimate the clinical rating scale (Movement Disorder Society- Unified Parkinson’s Disease Rating Scale part III; MDS-UPDRS-III). Twenty-nine features were found to significantly progress with time at group level. The Random Forest model revealed the most accurate estimation of the MDS-UPDRS-III among the seven models. The model estimations detected a statistically significant progression of the motor symptoms within 15 months when compared to the first visit, whereas the MDS-UPDRS-III did not capture any change. Wearable sensors and machine learning can track the motor symptom progression in people with PD better than the conventionally used clinical rating scales. The methods described in this study can be utilised complimentary to the clinical rating scales to improve the diagnostic and prognostic accuracy.

## Introduction

The progression of Parkinson’s disease (PD) is currently monitored using clinical rating scales which are used to assess the cardinal motor and non-motor symptoms. The current gold standard is the Movement Disorder Society-Unified Parkinson’s Disease Rating Scale (MDS-UPDRS)^1^.

Accurate assessment with rating scales depends on the clinician’s experience and interpretation may be complicated by inter-rater disagreement^2^. Rating scale data are coarse-grained, and on an ordinal rather than interval scale. Collectively, these factors introduce variability and delay the confident detection of progression; at the group level in clinical studies, they may restrict the types of statistical analysis that can be performed. Objective measures that are on a continuous interval scale would be highly desirable both for the assessment of individual patients in clinical practice and for measuring the efficacy of therapeutic interventions in clinical trials.

In recent years, wearable sensors have emerged as a promising tool for quantitative characterisation of motor status in PD^3,4^. The portability and affordability of wearable sensors make it possible to assess the spatio-temporal features of walking and balance in the laboratory or clinic, and remotely at the comfort of patients’ homes. The ultimate vision is to use these detailed and personalised kinematic measurements to individualise disease diagnostic and prognostic tools and measure the effectiveness of treatment.

Most wearable devices used to monitor PD patients output many objective numerical measures, leading to very large datasets. Not all features extracted by these devices are meaningful for clinical diagnosis and treatment. Analysis of high-dimensionality datasets usually requires initial feature reduction steps to avoid type 1 error while maintaining sensitivity. There is a need for the development of analysis techniques to extract clinically useful information from the high-dimensional data generated by these wearable sensors^5^.

Machine learning (ML) algorithms have been applied to data collected by wearable inertial measurement units (IMUs), which are combinations of triaxial accelerometers, gyroscopes, and magnetometers, often with multiple IMUs connected wirelessly in a body-area network^6,7^. ML algorithms may be trained with the clinical rating scales used in PD diagnosis as labels, using the movement features collected by IMUs when patients perform standard clinical assessment tasks^8,9^. Previous work from our lab and others has demonstrated that the analysis of IMU data can discriminate between healthy older adults, individuals with PD of different disease severity, and individuals with other Parkinsonian-like disorders, such as PSP^10–13^. Suitably-trained ML algorithms can identify freezers^14^ and fallers^15^, and detect signs of bradykinesia^16^ among PD individuals cross sectionally. Further, gait features extracted from the IMU data can be used prospectively to identify older adults at risk of developing PD^17^. Overall, these studies have shown that the combination of wearable device data with ML algorithms can duplicate clinical rating scales and discriminate between different disorders and phenotypes. The current longitudinal study investigates the use of kinematic features collected during walking and standing tasks to objectively track the progression of PD motor symptoms over time.

We have previously shown that kinematic features derived from the data collected by wearable devices placed on the participant’s trunk, wrists, and feet, together with machine learning algorithms can be used to track the progression of Progressive Supranuclear Palsy motor symptoms^18^. The current study aims to extend this knowledge, using walking and postural sway features derived from data collected by six IMUs, to identify the earliest signal of motor symptom progression in individuals with PD measured every three months, over a period of 18 months. First, seven different models based on ML algorithms with automatic feature selection and/or factorisation were investigated to validate the association of the wearable sensor-derived data with the MDS-UPDRS-III scale. Second, we aimed to establish whether the model-estimated scores could be used to track the progression of the PD motor symptoms. The hypotheses that were put forward were that a) the model-estimated scores based on wearable sensor-derived motor features could mimic the MDS-UPDRS-III score and b) the model-estimated scores would identify motor symptom progression earlier than the MDS-UPDRS-III score.

## Results

IMU data were obtained from 91 Individuals with idiopathic PD over a period of 18 months. Visits were scheduled at 3-month intervals (thus a total of 7 planned visits including baseline, i.e., the first visit). Participants were excluded if they missed more than 2 consecutive visits; this resulted in 74 going forward for further analysis. Demographics and clinical data are presented in Table 1.

**Table 1:** Demographics of PD participants.

PD demographics (*N* = 74) at Visit 1	Mean (standard deviation)
Age	64.6 (7.8)
Sex (Male/Female)	42/32
Height (cm)	172 (9.6)
Weight (kg)	75.9 (12.5)
Time since diagnosis (months)	56 (49)
Side of Symptom onset (R/L)	32/42
Dominant side (R/ L/ Ambidextrous)	65/7/2
MDS-UPDRS part III	24.4 (12.0)
MoCA	26.9 (2.3)

Demographics, clinical characteristics at Visit 1 and cognitive scores of PD patients. Mean values and standard deviation (in parenthesis). *MDS-UPDRS* Movement Disorders Society Unified Parkinson’s Disease Rating Scale, *MoCA* Montreal Cognitive Assessment.

### Automatic feature selection

Out of the total of 122 measured features (a list of which can be found in the Supplementary Table 1), the group means of 29 features were found to linearly increase or decrease significantly (*p* < 0.05) over time at a group level (Supplementary Fig. 1). These 29 “progressing features” were analysed further. Seven different regression analyses with automatic feature selection were performed and their results compared.

- A multivariate linear regression (LR) model using the two features with the most statistically significant progression over time was developed to estimate the MDS-UPDRS-III scale as its output (model 1). The features showing the most significant progression of their group means over time were the variabilities (in terms of standard deviation) of a) the terminal double support, and b) the swing phase, for the contralateral (to the side of motor symptom onset) lower limb.
- From the subset of the 29 progressing features, 6 were identified (i.e., an extra 4 added to the two features of model 1). This was achieved using forward feature selection, with early stopping (model 2). Model 2 was more accurate in estimating the MDS-UPDRS-III score in at least 5 visits than model 1.
- A Random Forest (RF) Regressor with all 29 progressing features as its input (model 3) was also investigated.
- Principal Component Analysis (PCA) was applied to a) the entire set of 122 features, and b) the subset of the 29 progressing features. This returned 31 and 10 factors, respectively, accounting for 90% of the total variance (See Supplementary Fig. 2). Again, both Linear Regression and Random Forest regression were used to estimate the MDS-UPDRS-III clinical rating scale, using the principal components as independent variables. This resulted in 4 models: a) model 4 using LR with 10 factors, b) model 5 using RF with 10 factors, c) model 6 using LR on 31 factors and d) model 7 using RF on 31 factors.

Table 2 shows the performance of each model used to estimate the MDS-UPDRS-III rating scale as its output. Performance metrics were calculated as the average Root Mean Square Error on the validation set using 5-fold cross validation analysis. Model 3 (RF regression to the progressing features) performed best with an average RMSE of 10.02 (Table 2) and was used to quantify motor symptom progression in all subsequent analyses.

**Table 2:** Model performance.

Set of features	Dimensionality reduction	Predictor	RMSE (std)
Progressing features (29 features)	Feature selection (2 features)	LR (model1)	11.86 (0.67)
	Feature selection (6 features)	LR (model2)	11.17 (0.80)
	**All progressing (29** features**)**	**RF (model3)**	**10.02 (0.88)**
	PCA (10 factors)	LR (model4)	11.25 (0.68)
		RF (model5)	10.92 (0.65)
Original features (122 features)	PCA (31 factors)	LR (model6)	10.80 (0.91)
		RF (model7)	10.32 (0.76)

The table shows the regression results when automatically selecting features.

*RMSE* Root Mean Square Error, *std* standard deviation.

**Fig. 1:**
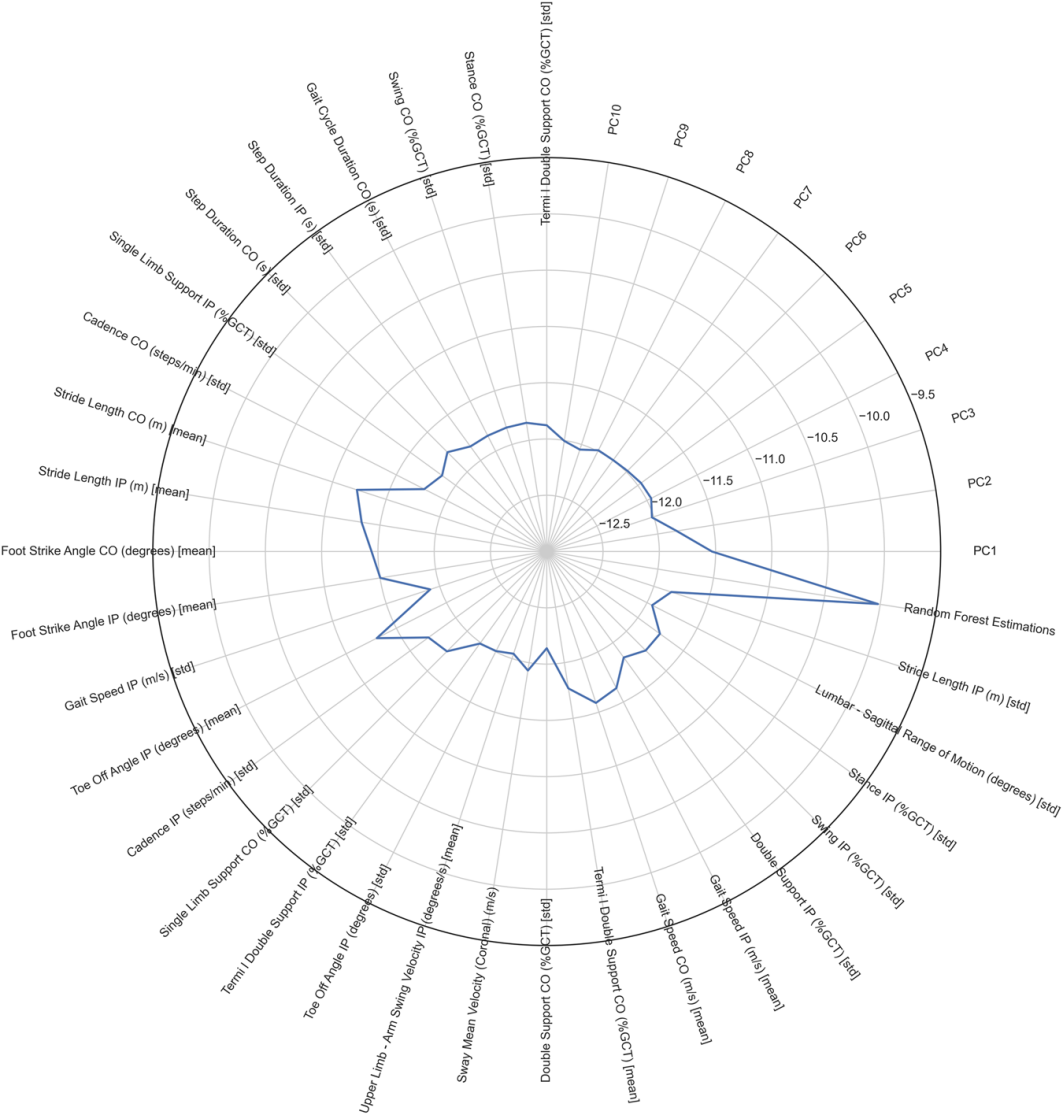
Radar plot of RMSE scores. The RMSE scores for each kinematic feature, principal component, and the model (Random Forest, model 3) estimations are shown. The Random Forest estimations estimated the MDS-UPDRS-III with the best accuracy.

To make sure that the model was able to estimate the clinical score more accurately than an individual feature on its own, the same cross-validation procedure was performed with a simple linear regression using each feature in turn as an MDS-UPDRS-III estimator. Figure 1 illustrates the estimation error (in terms of average RMSE across the 5-fold cross validation iterations) for each feature, Principal Component, and the RF (model 3). Among the 29 progressing features, stride length (RMSE; contralateral: 11.23, ipsilateral: 11.33), foot strike angle (RMSE; contralateral: 11.44, ipsilateral: 11.50) and the toe off angle (RMSE; ipsilateral: 11.30) were found to be more important independent predictors of MDS-UPDRS-III score.

### Actual and model estimated UPDRS progression

**Fig. 2:**
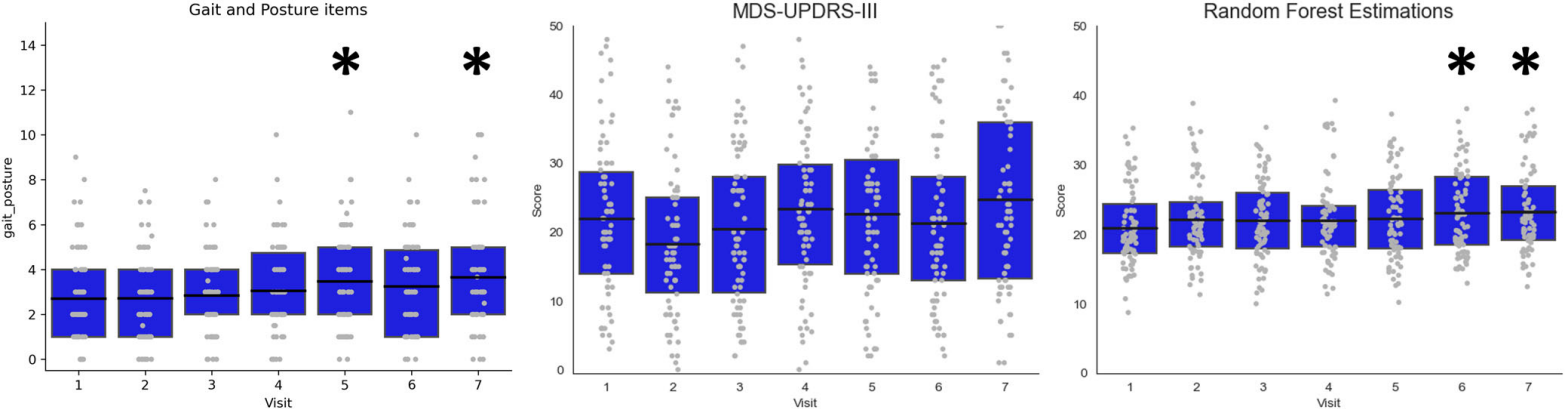
Progression of the clinical and model estimated scores. Boxplots illustrate the progression of the gait and posture items (left), the actual MDS-UPDRS-III (middle) and RF-estimated (right) MDS-UPDRS-III scores. Asterisks denote the significant adjusted pairwise comparisons to baseline.

Figure 2 shows the progression of the actual and the estimated MDS-UPDRS-III scores and an aggregate score of the actual MDS-UPDRS-III items that measure gait and posture functions (Gait, Freezing of Gait, Posture, Postural Stability). The mean scores, estimated by Model 3, demonstrated a monotonic increase from visit 1 to visit 7, in contrast to the means of the total MDS-UPDRS-III values assigned by the expert clinicians. Repeated measures analysis revealed that the model was able to provide an earlier signal of disease progression when compared to the actual MDS-UPDRS-III. A Friedman repeated measures test for related samples demonstrated that there was a significant change across visits for both the actual MDS-UPDRS-III values (χ^2^ = 28.83, *p* < 0.001), and the RF-estimated values (χ^2^ = 15.59, *p* = 0.016). Pairwise comparisons, after adjusting the significance threshold for multiple comparisons (Benjamini-Hochberg with 1% False Discovery Rate) showed that the actual MDS-UPDRS-III score did not increase at any visit when compared to baseline (Visit 1). In contrast, the RF-estimated MDS-UPDRS-III score was increased at V6 (*p* < 0.001) and V7 (*p* < 0.001) with respect to baseline. The group-median RF-estimations demonstrated a rate of change of 0.33 points per Visit (i.e., per 3 months).

The Gait and Posture items aggregate score progressed significantly but this progression was not purely monotonic, rather the score increased at V5 (*p* = 0.001) and V7 (*p* < 0.001) compared to the baseline but V6 was not significant. We further calculated the interquartile range (difference between the 25th and 75th percentile) for each visit for the clinical rating scale and the model estimations. The results emphasised that the MDS-UPDRS-III data are more variable compared to the RF estimations (MDS-UPDRS-III interquartile range per visit: V1 = 14.75, V2 = 13.75, V3 = 16.75, V4 = 14.5, V5 = 16.5, V6 = 15, V7 = 22.75; RF estimations: V1 = 7.14, V2 = 6.29, V3 = 7.98, V4 = 5.80, V5 = 8.37, V6 = 9.69, V7 = 7.68).

## Discussion

This study demonstrates a novel and objective method to quantify motor symptom progression in PD using a combination of wearable sensor data and ML algorithms. We applied automatic feature selection processes to the sensor data to cope with the plethora of measured kinematic features. The model that estimated the MDS-UPDRS-III score with the lowest RMSE (model 3) was then adopted to process the longitudinal sensor data from sequential visits. Results showed that the model was able to identify the worsening of PD motor symptoms over time from those data, unlike the actual MDS-UPDRS-III scores themselves.

The ability of ML methods to learn patterns from kinematic data and estimate disease severity has also been shown in previous studies in parkinsonian disorders^17–20^. In our study, the RF regressor (model 3) resulted in the lowest root mean square error (RMSE = 10.02) across the five cross-validation iterations, when compared to the other models (see Table 2) and all individual features (see Fig. 1). Random Forest regressors are able to account for the collinearities that exist in high-dimensional sensor datasets and have previously shown excellent discriminatory performance in similar situations^11,12,21^.

The primary aim of this study was to develop a method to track the progression of walking and postural sway kinematic features over time. Model 3 identified motor symptom progression as early as 15 months after baseline, while the clinical rating scale did not capture these signs of progression by the end of the period studied (see Fig. 2). Further, a computed sum of the gait and posture sub-items of the MDS-UPDRS revealed a progression which did not demonstrate monotonicity across visits. The better performance of the RF model to track the progression of the motor symptoms can be explained by the reduced variability of the modelled data, compared to the MDS-UPDRS-III. This was evidenced by the variability of the model estimations that was less than half of the variability manifested by the MDS-UPDRS-III at all visits (in terms of inter-quartile range). Effectively, the model output (i.e., estimated MDS-UPDRS-III score) increases monotonically from one visit to the next, whereas the actual MDS-UPDRS-III scores demonstrate visit-to-visit fluctuations and hence no clear evidence of progression of motor symptoms emerges. By decreasing the noise, the model results seem promising for the identification of an earlier motor progression signal in PD. The integration of wearable sensors, clinical rating scales and machine learning presents a promising method to assess the effectiveness of therapeutic interventions that target motor symptoms in PD.

The results of the current study also highlight the individual features that contribute most to an accurate estimate of the MDS-UPDRS-III-score: the angle of the foot at foot strike and toe off, and the stride length. Stride length has been previously reported to be a reduced in people with PD compared to healthy controls^22^. The foot strike angle measures the pitch angle of the foot at the point of initial contact with the ground, with smaller values indicating that the foot reaches the floor at a flatter angle. Importantly, foot strike angle decreases from one visit to the next (see Supplementary Fig. 1) suggesting that the foot pitch angle decreases as a function of disease duration, rendering patients increasingly prone to trips and falls^23^. A lower foot strike angle has been shown to be a discriminative characteristic of PD^24^ and a marker of disease severity^11^.

Out of the 29 features that showed statistically significant progression across visits, 19 reflect walking variability (measured in terms of standard deviation in this study; see Supplementary Fig. 1). Step to step variability has been previously shown to scale with disease severity in PD^25^ while Deep Brain Stimulation can reduce it^26^. Furthermore, step to step variability is also an important predictor of falls^15^. Despite their significant progression across visits variability measures were not found to correlate well with the MDS-UPRDS-III score (Fig. 1). This means that although walking variability may be an important feature to identify motor symptom progression in PD, it may not be routinely captured during clinical examination and without the help of digital technology. From the list of postural sway features, the mediolateral sway velocity (coronal plane) was the single postural feature that progressed significantly across visits and was therefore included in the model as an independent predictor. Moreover, mediolateral sway velocity has been previously shown to be an important biomarker of falls in PD^27^.

Kinematic features collected by wearable devices and analysed using a well-known Machine Learning algorithm can provide early signs of PD motor symptom progression, enabling the assessment of the effectiveness of medical treatment. We propose the methodology presented in this study as a complementary tool for assessment of PD patients in the clinic.

## Methods

91 Individuals with PD were recruited through the Oxford Quantification in Parkinsonism (OxQUIP) study, conducted at the John Radcliffe Hospital, Oxford, UK. The study was approved by a research ethics committee and the Health Research Authority (REC 16/SW/0262). All participants were informed about the study’s aims and protocols, and gave their informed written consent to participate in the study. Participants were included if they a) were diagnosed with Parkinson’s Disease, b) received anti-parkinsonian medication, c) had no major musculo-skeletal problems that precluded them from walking or standing and d) could walk and stand unassisted during the clinical tests. All participants were asked to visit the lab once every three months over a period of 18 months, completing a total of 7 visits. Participants were also tested using the Montreal Cognitive Assessment (MoCA) test when entering the study, to ensure that they were not demented (MoCA > 24) at the time of giving their consent. All patients were receiving antiparkinsonian medication at the time of their first visit to the lab and continued receiving their medication up to the end of the study.

### Apparatus and task

Participants were rated using the MDS-UPDRS-III score (motor part of the overall MDS-UPDRS score). They were subsequently asked to perform two movement tasks, to assess walking and postural sway. The walking task lasted for 2 minutes and was performed on a straight level surface, in a 15-metre-long corridor, making turns when necessary. To measure postural sway, the participants were instructed to stand still for 30 seconds with their eyes closed. A footplate was initially placed between each participant’s feet to ensure standardised inter-foot distance across participants and removed immediately before starting the data collection for each task. An experienced clinical researcher was standing or walking on the participant’s side for safety reasons, and to ensure eye closure during the postural sway task.

For both movement tasks, an array of 6 IMU sensors (OpalTM, APDM, Portland, Oregon, USA) was used to collect the kinematic data. The sensors were placed on both wrists and feet, the sternum, and the lumbar region. Each sensor provides triaxial accelerometer, gyroscope and magnetometer data at a sampling frequency of 128 Hz.

### Data analysis

All analyses were performed using custom software written in Python (v3.8).

**Fig. 3:**
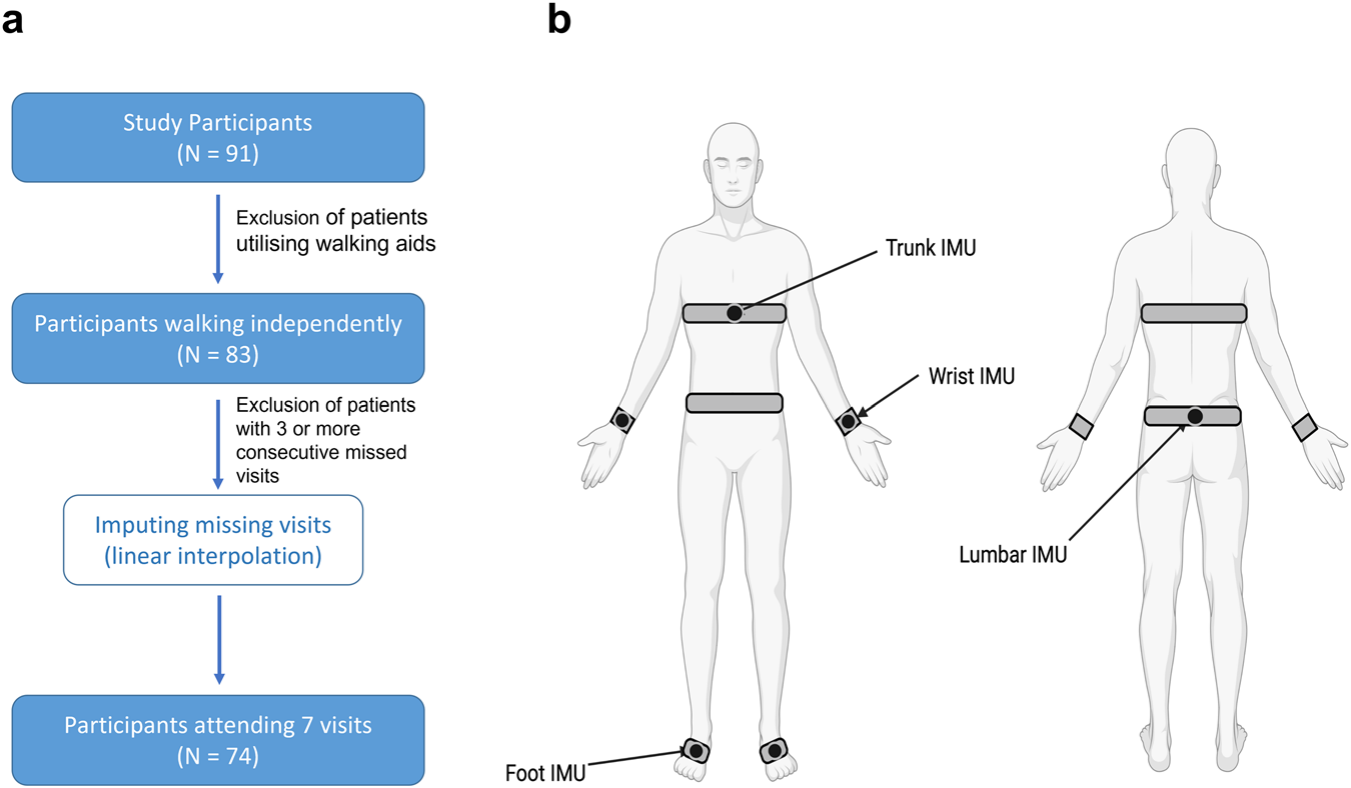
Experimental setup. **a** Schematic diagram illustrating the participant selection process. **b** Illustration of the 6 IMU sensor placement (Image created with BioRender.com). IMU Inertial Measurement Unit.

Several participants dropped out during the study or missed one or more visits. Participants with more than two consecutive missed visits were excluded. If a participant missed only one or two visits, imputation was performed using linear interpolation, i.e., data were imputed considering the feature values for the same participant at the previous and the next available visit. This resulted in a total number of 74 participants being included in the analysis. Figure 3 schematically illustrates the participant inclusion criteria for the study.

### Pre-processing

From the MDS-UPDRS-III score, items 3.1 and 3.2 (measuring Speech and Facial expression, respectively) were omitted because these functions are less related to walking and swaying. The total MDS-UPDRS-III was calculated as the sum of the values of all other items. Furthermore, an aggregate score of the posture and walking features was calculated, as the sum of the following items: 3.10 “Gait”, 3.11 “Freezing of Gait”, 3.12 “Postural Stability” and 3.13 “Posture”.

The MobilityLab software extracted 122 kinematic features for both walking and postural sway tasks. As a first step in pre-processing, stride length features (mean and variability) were normalised by the participant’s height, expressed in metres. Additionally, the *right* and *left* limb-specific features were relabelled *Ipsilateral* and *Contralateral*, depending on the participants’ self-reported side of symptom onset. Ipsilateral refers to the side which developed symptoms first.

### Dimensionality reduction and feature selection

**Fig. 4:**
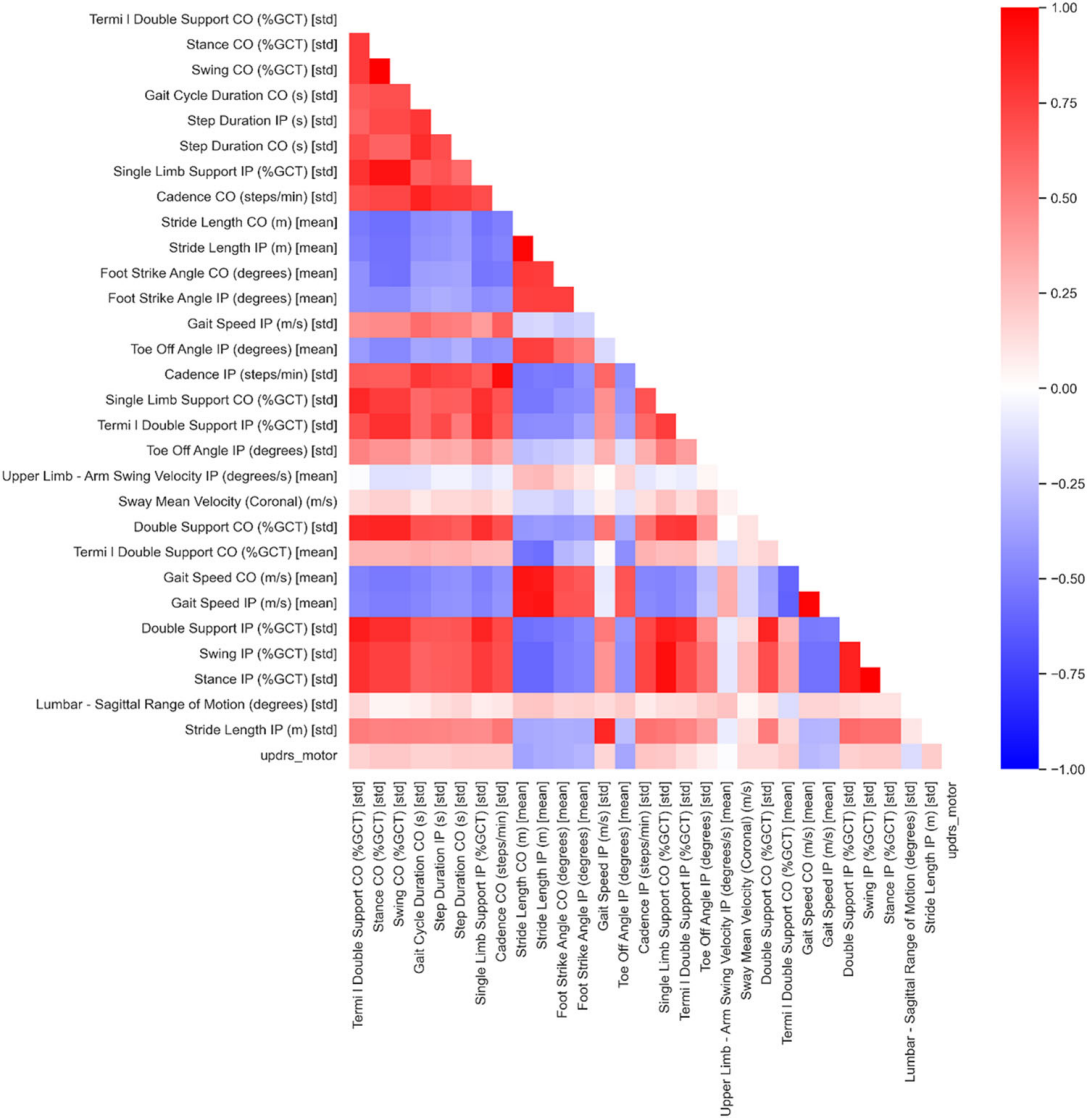
Correlation heatmap. The correlations among the 29 features and the MDS-UPDRS-III found to linearly increase or decrease significantly (*p* < 0.05) over time are shown. Blue and red coloured cells illustrate negative and positive correlation respectively.

The high dimensionality of kinematic features and the collinearities in the dataset (Fig. 4), necessitated a feature selection step. As a first step of the dataset dimensionality reduction, the 122 feature values were averaged across participants for each visit. The features for which the group means were found to linearly increase or decrease significantly (Linear Regression *p* < 0.05) across the seven visits were considered to be “progressing features”. 29 progressing features were stored for further analysis.

**Fig. 5:**
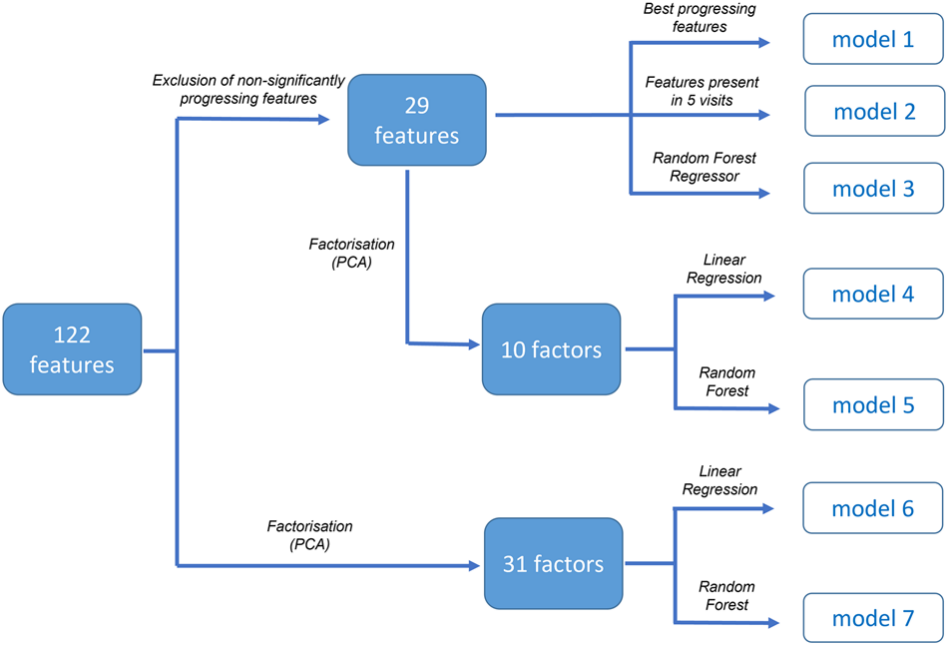
Feature selection and analysis pipeline. Different feature selection, factorisation and modelling strategies were investigated resulting in 7 different models. The model that estimated MDS-UPDRS-III more accurately (with the smallest RMSE) was selected for further analysis.

Different automatic feature selection strategies and models were subsequently investigated in order to select the combination that performed best, based on the Root Mean Square Error (RMSE) derived by cross-validation analysis. Figure 5 illustrates the steps of dimensionality reduction.

First, the two most significant features shown to progress with time (i.e., regress linearly across visits) based on the group average were used to estimate the MDS-UPDRS-III score, using a multivariate linear regression.

Second, a forward feature selection with early stopping was used as described in our previous study^18^. Briefly, the dataset was split into a training set composed of the wearable sensor data acquired in 6 out of the 7 visits, and a validation set corresponding to the data acquired during the remaining visit. This meant that there were 7 training datasets (consisting of data acquired in 6 out of 7 visits) and 7 corresponding validation datasets (consisting of the data acquired during the remaining visit in each case). For each of the 7 iterations, a simple linear regression algorithm was used to select those features that gave the most accurate estimation of the MDS-UPDRS-III score. Each of the 7 models was initialised with the single feature which had previously shown the best progression across visits (lowest *p*-value: the terminal double support of the contralateral limb (% of gait cycle time). The algorithm then iterated through the list of the remaining progressing features and added the one that reduced the RMSE by the greatest amount. This process was repeated 7 times, until all visits served as a validation set once. Effectively, this step provided a list of features to estimate the MDS-UPDRS-III for each visit. The features that were present in at least 5 of the 7 visits were stored as important features to be used in model validation.

Third, Principal Component Analysis (PCA) was introduced as a procedure to reduce the dimensionality of the original datasets using fewer uncorrelated factors, called principal components. PCA was applied both to the entire set of 122 features, and on the 29 progressing features, resulting in two sets of principal components. We selected the number of principal components that explained 90% of the total variance of each dataset.

Finally, as a part of a Random Forest Regressor the relative importance of the features is determined based on the amount of impurity (in terms of squared error) reduction in each decision tree. This is then averaged across the ensemble decision trees to determine the eventual features importance. The entire set of 29 progressing features was used and no further selection process performed prior to RF regression because this algorithm is known to deal well with high-dimensionality datasets and collinearities among the features.

### Cross validation

A five-fold repeated cross-validation process was used to evaluate each model and select the one that most accurately estimated the MDS-UPDRS-III values. The entire dataset (74 participants, 7 visits) was randomly split into 5 subsets. Four subsets (80% of the data) were subsequently used as the training set, while the remaining subset (20%) served as the validation set. This process was repeated 5 times, and so all sub-sets were each used as part of the training and the validation set once. The model that performed best in estimating MDS-UPDRS-III scores (model 3) was selected to analyse the dataset longitudinally.

### Statistics

The normality of distribution was tested using the Shapiro-Wilks test and the model-estimated MDS-UPDRS-III score was found to be non-normally distributed at most visits (Shapiro-Wilks for MDS-UPDRS-III: V1 = 0.067, V2 = 0.021, V3 = 0.036, V4 = 0.818, V5 = 0.184, V6 = 0.005, V7 = 0.013; Shapiro-Wilks for RF model estimations: V1 = 0.015, V2 = 0.008, V3 = 0.769, V4 = 0.001, V5 = 0.142, V6 = 0.008, V7 = 0.003). The inter-quartile range was calculated for each visit as means to estimate the noise for both MDS-UPDRS-III and the model estimations. A Friedman test for related samples was therefore used to address whether the model-estimated MDS-UPDRS-III values exhibited progression across visits. Pairwise comparisons were further assessed between each visit and the baseline (i.e., visit 1), using the Wilcoxon signed rank test. A Benjamin-Hochberg correction for multiple comparisons was applied using a 1% False Discovery Rate (FDR).

### Reporting summary

Further information on research design is available in the Nature Research Reporting Summary linked to this article.

### Supplementary information

Supplementary Material

Reporting Summary

## Data Availability

Original data presented in this paper, is from the ongoing OxQUIP study and cannot be shared until completion of the whole study and full dissemination of results. This is expected to become possible within 24 months from the end of the study. Qualified researchers will be able to contact the Principal Investigator at the University of Oxford.

## Code availability

The underlying code for this study [and training/validation datasets] is not publicly available but may be made available to qualified researchers on reasonable request from the corresponding author.
