# Supplementary material for: Identification of motor progression in Parkinson’s disease using wearable sensors and machine learning
Source: NPJ Parkinsons Dis. 2023 Oct 7;9:142. doi: 10.1038/s41531-023-00581-2 (PMC10560243; doi:10.1038/s41531-023-00581-2)

Supplementary Material

Supplementary Table 1 ***Kinematic features extracted by the IMU software****. IP = ipsilateral side, CO = Contralateral side, std = standard deviation, RMS = Root Mean Square, AP = Antero-Posterior, ML = Medio-Lateral*

**Activity**	**Feature**	**Number of parameters**
Gait	Cadence	4 (IP mean & std, CO mean & std)
	Double Support	4 (IP mean & std, CO mean & std)
	Elevation at Midswing	4 (IP mean & std, CO mean & std)
	Gait Cycle Duration	4 (IP mean & std, CO mean & std)
	Gait Speed	4 (IP mean & std, CO mean & std)
	Lateral Step Variability	2 (IP, CO)
	Circumduction	4 (IP mean & std, CO mean & std)
	Foot Strike Angle	4 (IP mean & std, CO mean & std)
	Toe Off Angle	4 (IP mean & std, CO mean & std)
	Single Limb Support	4 (IP mean & std, CO mean & std)
	Stance	4 (IP mean & std, CO mean & std)
	Step Duration	4 (IP mean & std, CO mean & std)
	Stride Length	4 (IP mean & std, CO mean & std)
	Swing Phase (%GCT)	4 (IP mean & std, CO mean & std)
	Terminal Double Support	4 (IP mean & std, CO mean & std)
	Toe Out Angle	4 (IP mean & std, CO mean & std)
	Lumbar Range of Motion	6 (Coronal mean & std; Sagittal mean & std; Transverse mean & std)
	Trunk Range of Motion	6 (Coronal mean, std; Sagittal mean, std; Transverse mean, std)
	Arm Swing Velocity	4 (IP mean & std, CO mean & std)
	Arm Range of Motion	4 (IP mean & std, CO mean & std)
Turns	Turn Angle	2 (mean & std)
	Turn Duration	2 (mean & std)
	Turn Velocity	2 (mean & std)
	Turn – steps	2 (mean & std)
Sway	Sway 95% ellipse	3 (Radius ax1, ax2; Rotation)
	Sway Area	7 (Area; RMS AP, ML, total; Range AP, ML, total)
	Sway Frequency	6 (Centroidal AP, ML, total; Dispersion AP, ML, total)
	Jerk	3 (AP, ML, total)
	Velocity	3 (AP, ML, total)
	Path Length	3 (AP, ML, total)
	Sway angles	7 (Ellipse AP, ML; Ellipse Rotation; Sway area; RMS AP, ML, total)

Supplementary Figure 1 ***Significantly Progressing Features****. The 29 features progressed significantly based on their group means are presented in order of significance. The progression of the MDS-UPDRS motor part is illustrated (bottom-right).*

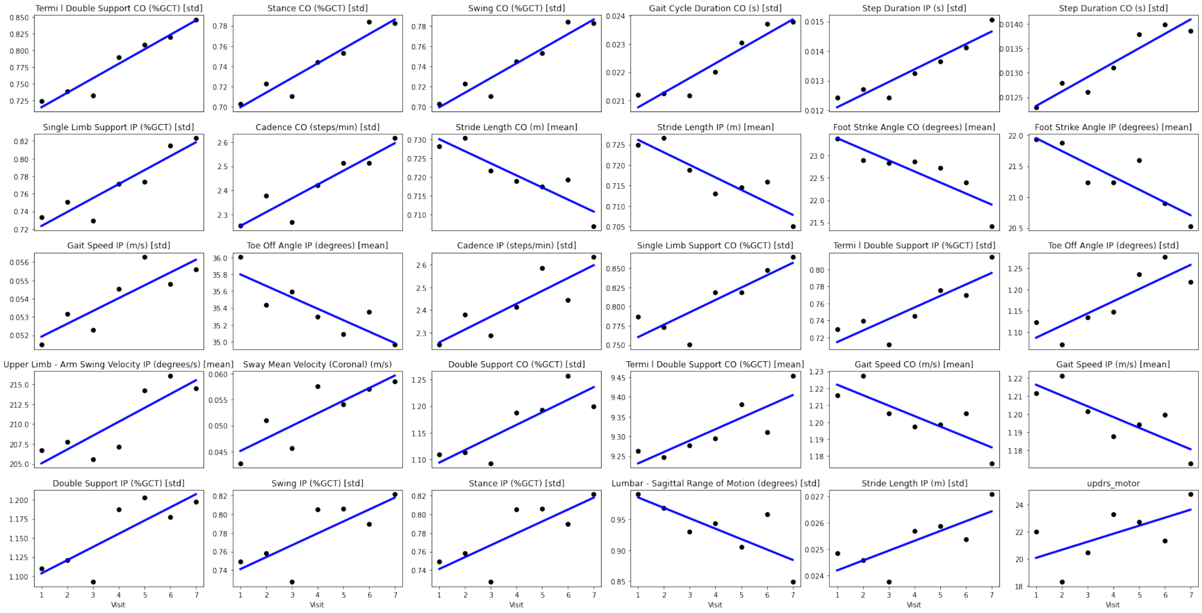

Supplementary Figure 2 ***Principal component analysis (PCA) explained Variance.*** *PCA applied to the entire dataset (122 features) and the 29 progressing features.*

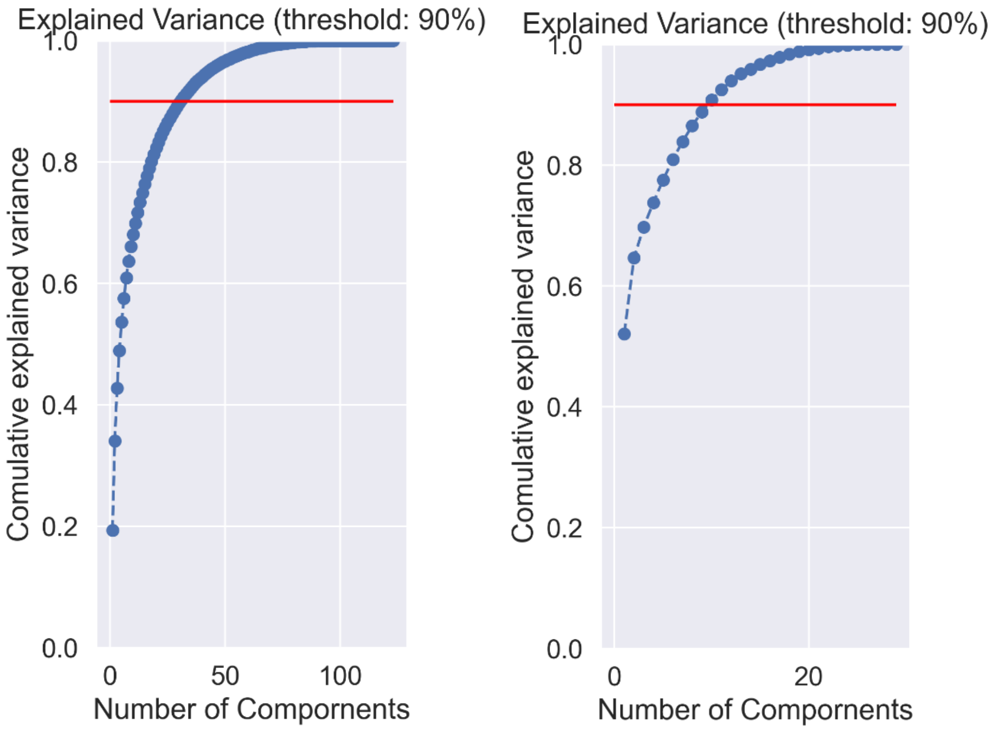

Supplement: Supplementary file 1 — Supplementary Material [file 41531_2023_581_MOESM1_ESM.pdf]
