# Supplementary material for: Identification of motor progression in Parkinson’s disease using wearable sensors and machine learning
Source: NPJ Parkinsons Dis. 2023 Oct 7;9:142. doi: 10.1038/s41531-023-00581-2 (PMC10560243; doi:10.1038/s41531-023-00581-2)
Supplement: Supplementary file 2 — Reporting Summary [file 41531_2023_581_MOESM2_ESM.pdf]

nature portfolio

Corresponding author(s): Chrystalina Antoniades

Last updated by author(s): 13/09/2023

# Reporting Summary

Nature Portfolio wishes to improve the reproducibility of the work that we publish. This form provides structure for consistency and transparency in reporting. For further information on Nature Portfolio policies, see our Editorial Policies and the Editorial Policy Checklist.

## Statistics

For all statistical analyses, confirm that the following items are present in the figure legend, table legend, main text, or Methods section.

| n/a | Confirmed | |
|---|---|---|
| ☐ | ☒ | The exact sample size (*n*) for each experimental group/condition, given as a discrete number and unit of measurement |
| ☐ | ☒ | A statement on whether measurements were taken from distinct samples or whether the same sample was measured repeatedly |
| ☐ | ☒ | The statistical test(s) used AND whether they are one- or two-sided<br>*Only common tests should be described solely by name; describe more complex techniques in the Methods section.* |
| ☐ | ☒ | A description of all covariates tested |
| ☐ | ☒ | A description of any assumptions or corrections, such as tests of normality and adjustment for multiple comparisons |
| ☐ | ☒ | A full description of the statistical parameters including central tendency (e.g. means) or other basic estimates (e.g. regression coefficient) AND variation (e.g. standard deviation) or associated estimates of uncertainty (e.g. confidence intervals) |
| ☐ | ☒ | For null hypothesis testing, the test statistic (e.g. *F*, *t*, *r*) with confidence intervals, effect sizes, degrees of freedom and *P* value noted<br>*Give P values as exact values whenever suitable.* |
| ☒ | ☐ | For Bayesian analysis, information on the choice of priors and Markov chain Monte Carlo settings |
| ☒ | ☐ | For hierarchical and complex designs, identification of the appropriate level for tests and full reporting of outcomes |
| ☒ | ☐ | Estimates of effect sizes (e.g. Cohen's *d*, Pearson's *r*), indicating how they were calculated |

*Our web collection on statistics for biologists contains articles on many of the points above.*

## Software and code

Policy information about availability of computer code

| | |
|---|---|
| Data collection | Data were collected using the proprietary MobilityLab software that is sold with the APDM system, as described in the methods section |
| Data analysis | Analysis was performed using custom software written in Python |

For manuscripts utilizing custom algorithms or software that are central to the research but not yet described in published literature, software must be made available to editors and reviewers. We strongly encourage code deposition in a community repository (e.g. GitHub). See the Nature Portfolio guidelines for submitting code & software for further information.

## Data

Policy information about availability of data

All manuscripts must include a data availability statement. This statement should provide the following information, where applicable:

- Accession codes, unique identifiers, or web links for publicly available datasets
- A description of any restrictions on data availability
- For clinical datasets or third party data, please ensure that the statement adheres to our policy

Data could be provided upon reasonable request

## Research involving human participants, their data, or biological material

Policy information about studies with human participants or human data. See also policy information about sex, gender (identity/presentation), and sexual orientation and race, ethnicity and racism.

| | |
|---|---|
| Reporting on sex and gender | Gender is reported in the demographic table and was self reported. It did not form part of the analysis. |
| Reporting on race, ethnicity, or other socially relevant groupings | These were not relevant variables in this study and there is no mention of them in the manuscript. |
| Population characteristics | Age is reported in the demographics table for the group as a whole but did not form art of the analysis. The patients all had idiopathic PD (not a genetic form of PD) and were not genotyped. |
| Recruitment | Recruitment was through a number of methods:<br>• Invitation to participate at the time of attendance at a specialist movement disorders clinic<br>• Leaflets available in the neuroscience outpatients department, West Wing, John Radcliffe Hospital, Oxford<br>• Advertisement via Parkinson's UK website and PSP association website, and association newsletters<br>• Advertisement via the Oxford branch of Parkinson's UK<br>• Advertisement on our research group's website<br>• Advertisement via relevant forums through the National Institute of Healthcare Research. |
| Ethics oversight | This study has been reviewed and given favourable opinion by South West – Cornwall and Plymouth Research Ethics Committee. |

Note that full information on the approval of the study protocol must also be provided in the manuscript.

# Field-specific reporting

Please select the one below that is the best fit for your research. If you are not sure, read the appropriate sections before making your selection.

☒ Life sciences ☐ Behavioural & social sciences ☐ Ecological, evolutionary & environmental sciences

For a reference copy of the document with all sections, see nature.com/documents/nr-reporting-summary-flat.pdf

# Life sciences study design

All studies must disclose on these points even when the disclosure is negative.

| | |
|---|---|
| Sample size | Sample size for this observational cohort study was 91 and was determined by practicality. The study is highly intensive, requiring detailed assessments of each participant on a 3 monthly basis, and the sample size was therefore limited to the number who could be assessed in each 3 month period. |
| Data exclusions | No data were excluded. |
| Replication | Cross validation was performed. This is a standard technique in machine learning. |
| Randomization | Not applicable, this is an observational cohort study. |
| Blinding | Not applicable. |

# Reporting for specific materials, systems and methods

We require information from authors about some types of materials, experimental systems and methods used in many studies. Here, indicate whether each material, system or method listed is relevant to your study. If you are not sure if a list item applies to your research, read the appropriate section before selecting a response.

## Materials & experimental systems

| n/a | Involved in the study |
|---|---|
| ☒ | ☐ Antibodies |
| ☒ | ☐ Eukaryotic cell lines |
| ☒ | ☐ Palaeontology and archaeology |
| ☒ | ☐ Animals and other organisms |
| ☐ | ☒ Clinical data |
| ☒ | ☐ Dual use research of concern |
| ☒ | ☐ Plants |

## Methods

| n/a | Involved in the study |
|---|---|
| ☒ | ☐ ChIP-seq |
| ☒ | ☐ Flow cytometry |
| ☒ | ☐ MRI-based neuroimaging |

# Clinical data

Policy information about clinical studies

All manuscripts should comply with the ICMJE guidelines for publication of clinical research and a completed CONSORT checklist must be included with all submissions.

| | |
|---|---|
| Clinical trial registration | This is an observational study, not a clinical trial. |
| Study protocol | Not a clinical trial. The procedures are fully described in the methods. |
| Data collection | 3 monthly visits at the John Radcliffe Hospital, Oxford, UK |
| Outcomes | Primary and secondary outcomes not applicable terms as this was not a clinical trial. The measures used are fully described in the methods. |
